# Supplementary material for: Beyond benchmarking: an expert-guided consensus approach to spatially aware clustering
Source: bioRxiv. 2025 Jun 27:2025.06.23.660861. Preprint. [Version 1] doi: 10.1101/2025.06.23.660861 (PMC12262716; doi:10.1101/2025.06.23.660861)
Supplement: Supplementary file 1 [file NIHPP2025.06.23.660861v1-supplement-3.pdf]

bioRxiv preprint doi: https://doi.org/10.1101/2025.06.23.660861; this version posted June 27, 2025. The copyright holder for this preprint (which was not certified by peer review) is the author/funder, who has granted bioRxiv a license to display the preprint in perpetuity. It is made available under aCC-BY 4.0 International license.

Supplementary Materials

Supplementary Figures

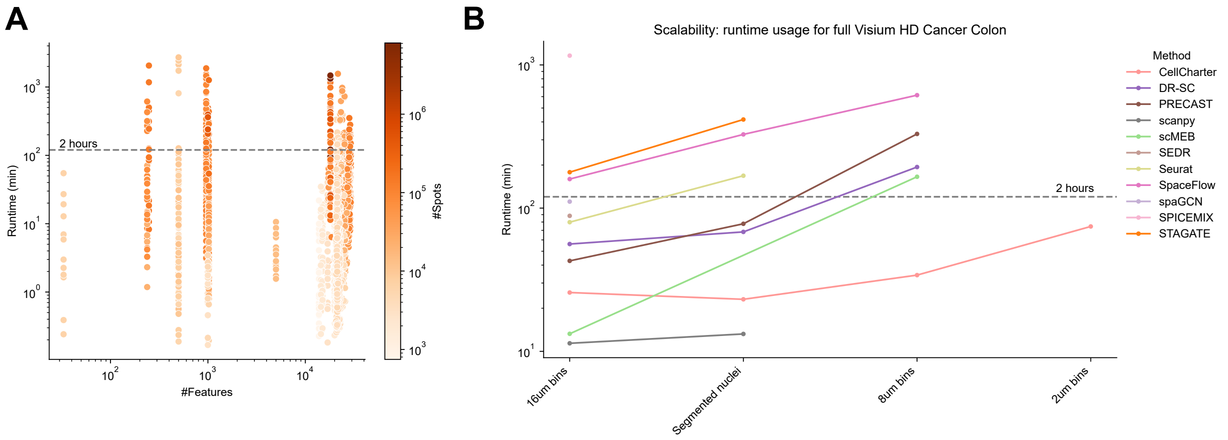

Figure S1: **A**. CPU runtime vs the number of features, colored by the number of spots/cells in the dataset. **B**. CPU runtime of methods on the full Visium HD colorectal cancer dataset across various bin sizes.

bioRxiv preprint doi: https://doi.org/10.1101/2025.06.23.660861; this version posted June 27, 2025. The copyright holder for this preprint (which was not certified by peer review) is the author/funder, who has granted bioRxiv a license to display the preprint in perpetuity. It is made available under aCC-BY 4.0 International license.

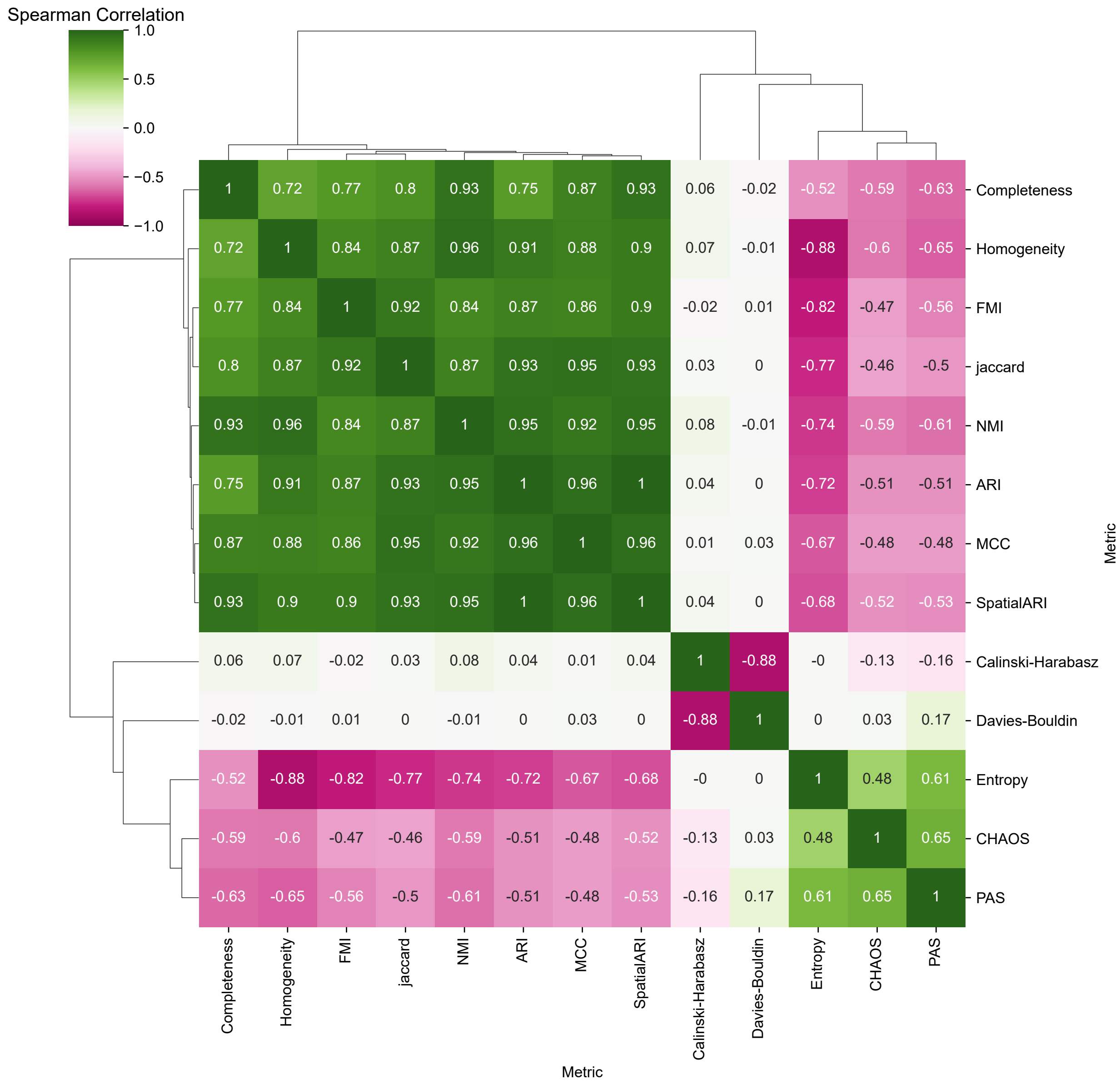

Figure S2: Correlation matrix of different metrics across all methods and samples from the LIBD Visium dataset.

bioRxiv preprint doi: https://doi.org/10.1101/2025.06.23.660861; this version posted June 27, 2025. The copyright holder for this preprint (which was not certified by peer review) is the author/funder, who has granted bioRxiv a license to display the preprint in perpetuity. It is made available under aCC-BY 4.0 International license.

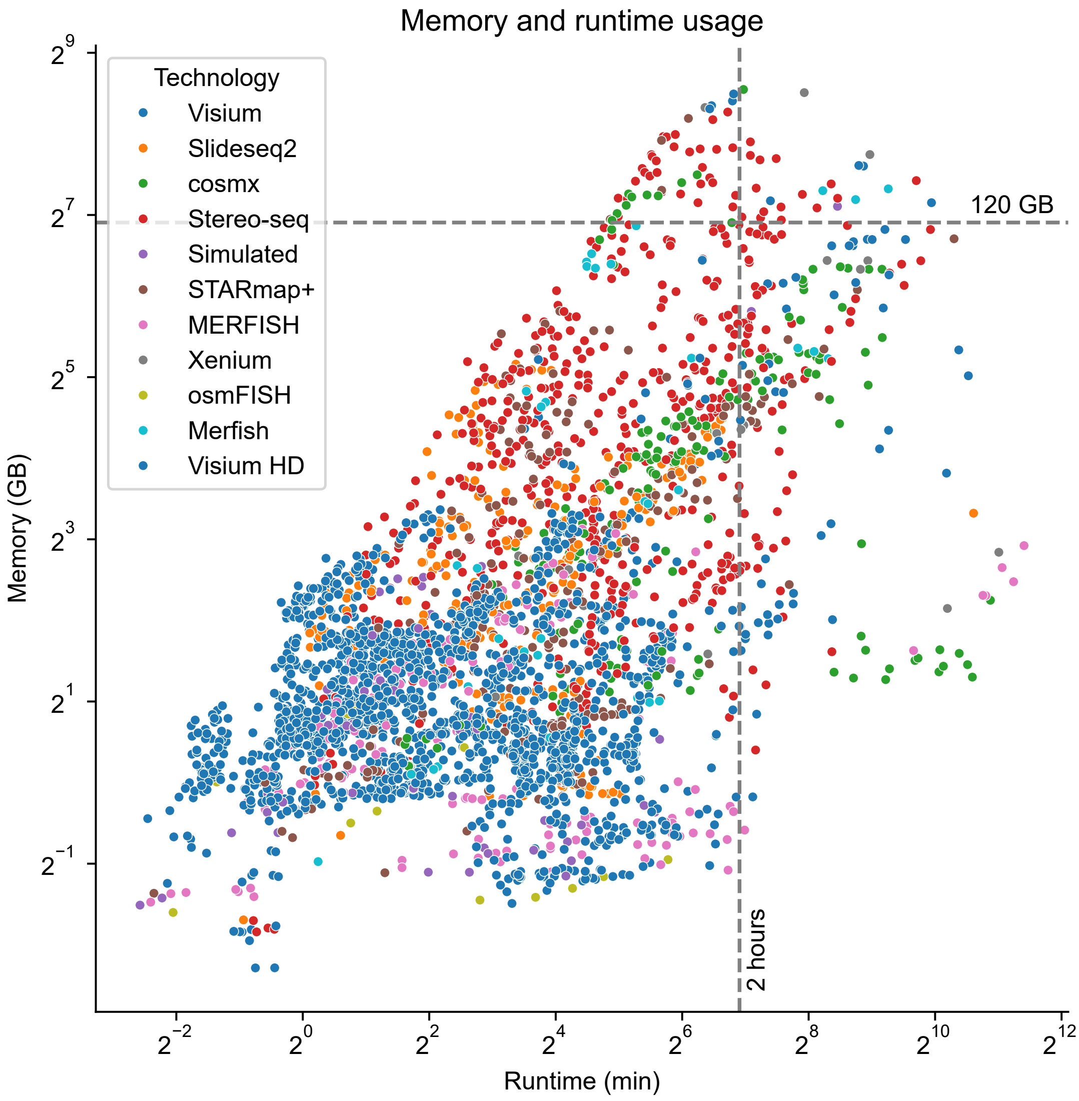

Figure S3: Memory usage vs CPU runtime, colored by the technology of the dataset.

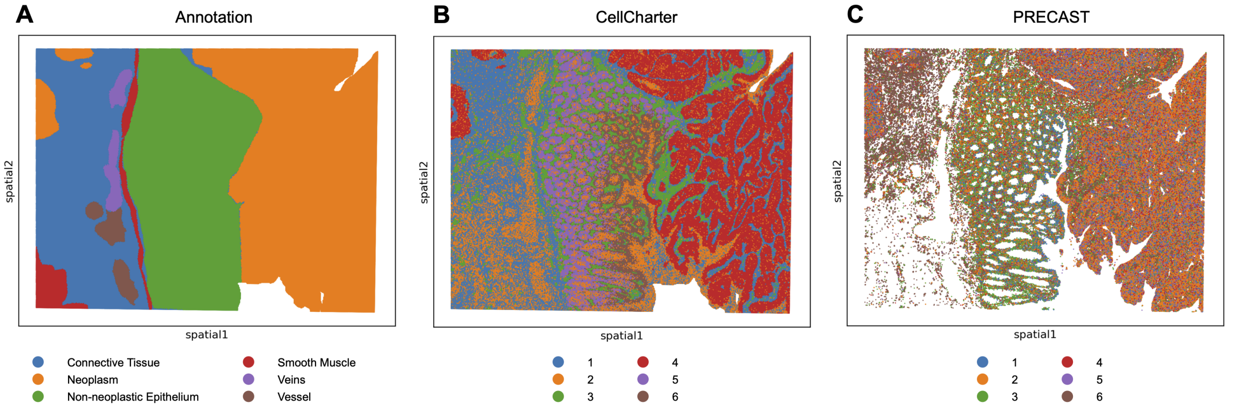

Figure S4: Spatial plots of the cropped Visium HD colon cancer dataset with 2 m bins, colored by **A**. ground truth, **B**. CellCharter clusters, and **C**. PRECAST clusters.

bioRxiv preprint doi: https://doi.org/10.1101/2025.06.23.660861; this version posted June 27, 2025. The copyright holder for this preprint (which was not certified by peer review) is the author/funder, who has granted bioRxiv a license to display the preprint in perpetuity. It is made available under aCC-BY 4.0 International license.

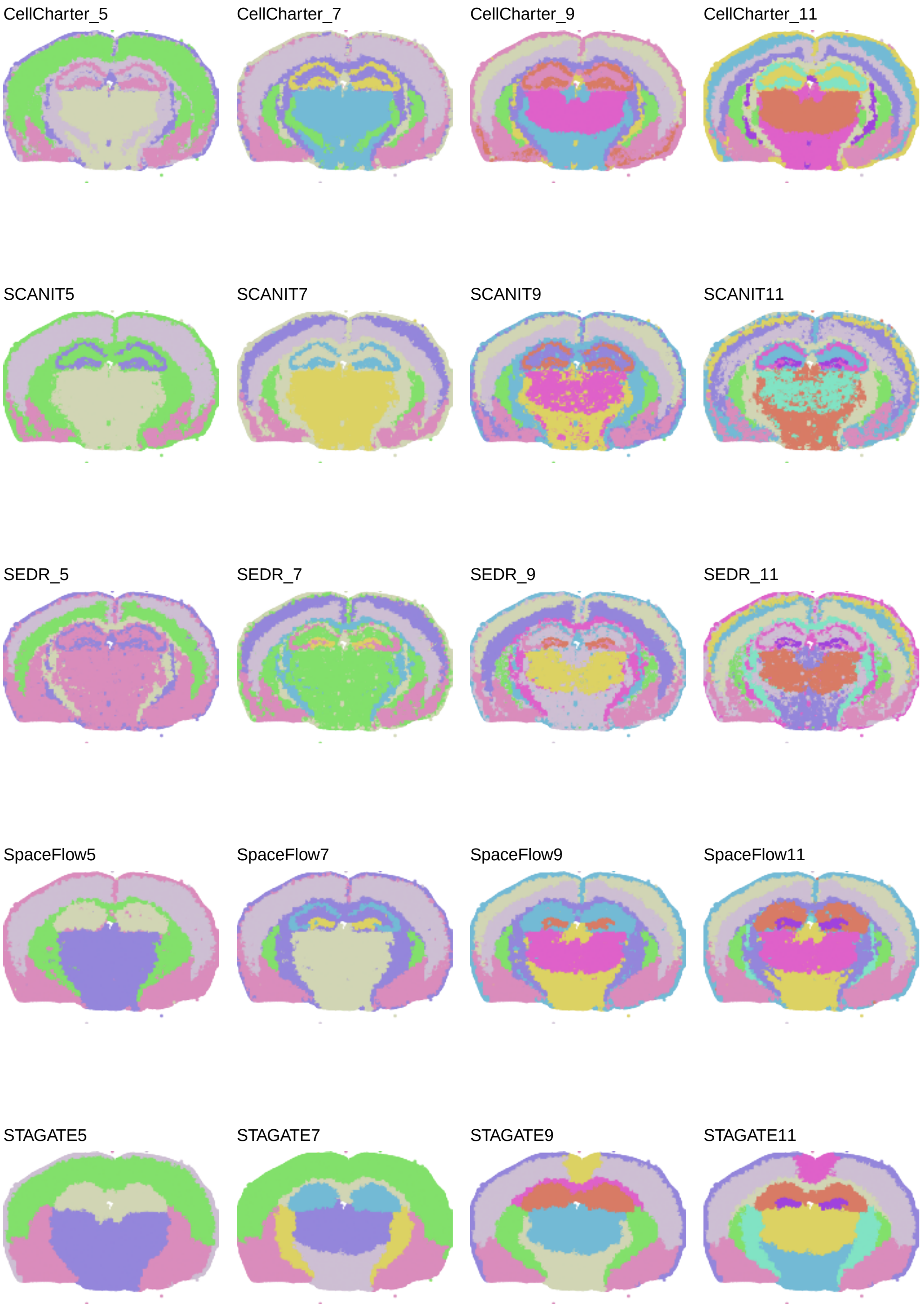

Figure S5: Spatial plots of Xenium mouse brain dataset cluster sweep results.

bioRxiv preprint doi: https://doi.org/10.1101/2025.06.23.660861; this version posted June 27, 2025. The copyright holder for this preprint (which was not certified by peer review) is the author/funder, who has granted bioRxiv a license to display the preprint in perpetuity. It is made available under aCC-BY 4.0 International license.

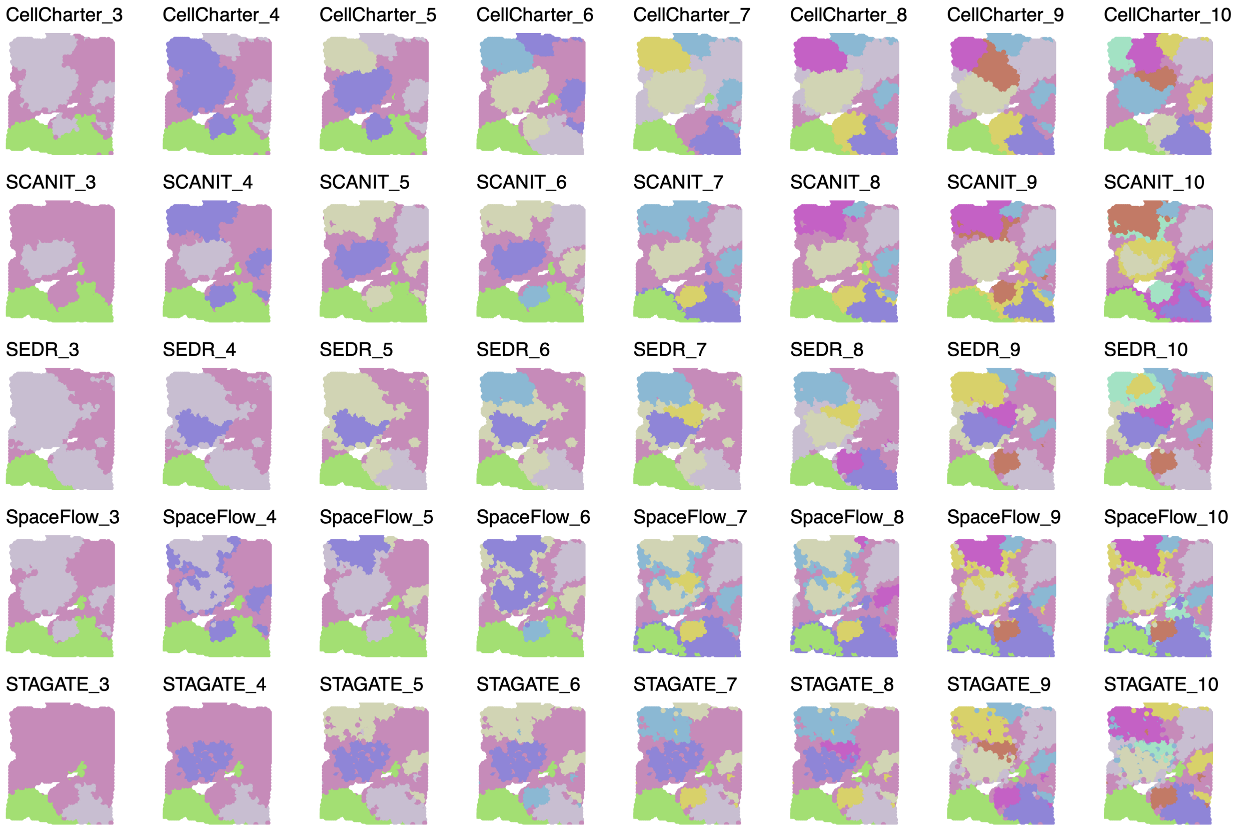

Figure S6: Spatial plots of Visium breast cancer dataset cluster sweep results.

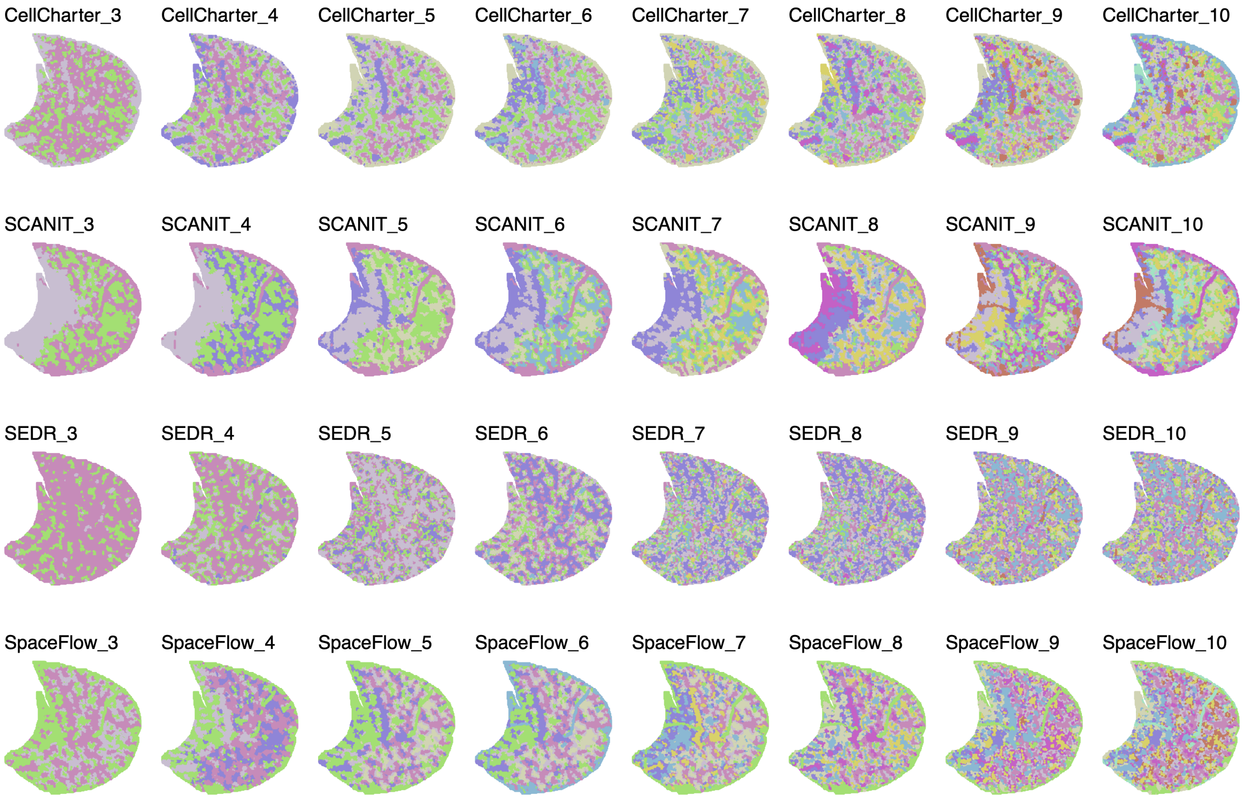

Figure S7: Spatial plots of Stereo-seq mouse liver dataset cluster sweep results with all genes.

bioRxiv preprint doi: https://doi.org/10.1101/2025.06.23.660861; this version posted June 27, 2025. The copyright holder for this preprint (which was not certified by peer review) is the author/funder, who has granted bioRxiv a license to display the preprint in perpetuity. It is made available under aCC-BY 4.0 International license.

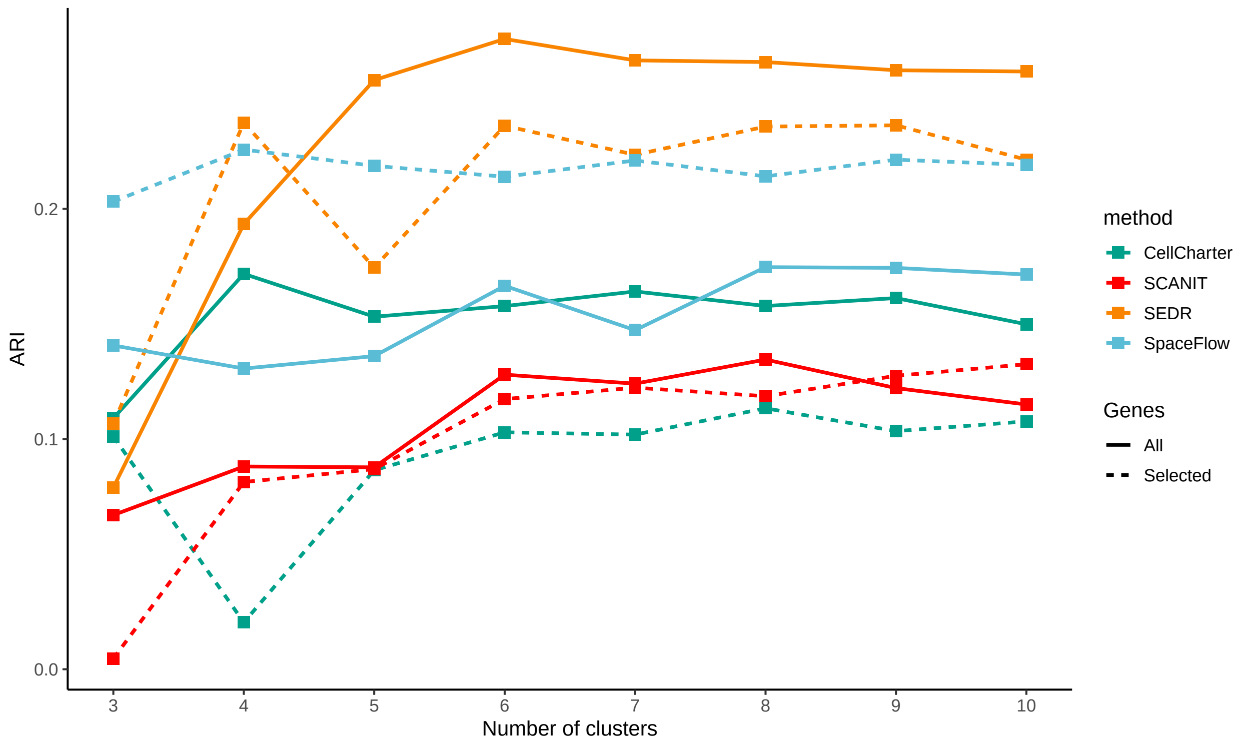

Figure S8: ARI results of Stereo-seq mouse liver datasets with all genes or selected marker genes.

bioRxiv preprint doi: https://doi.org/10.1101/2025.06.23.660861; this version posted June 27, 2025. The copyright holder for this preprint (which was not certified by peer review) is the author/funder, who has granted bioRxiv a license to display the preprint in perpetuity. It is made available under aCC-BY 4.0 International license.

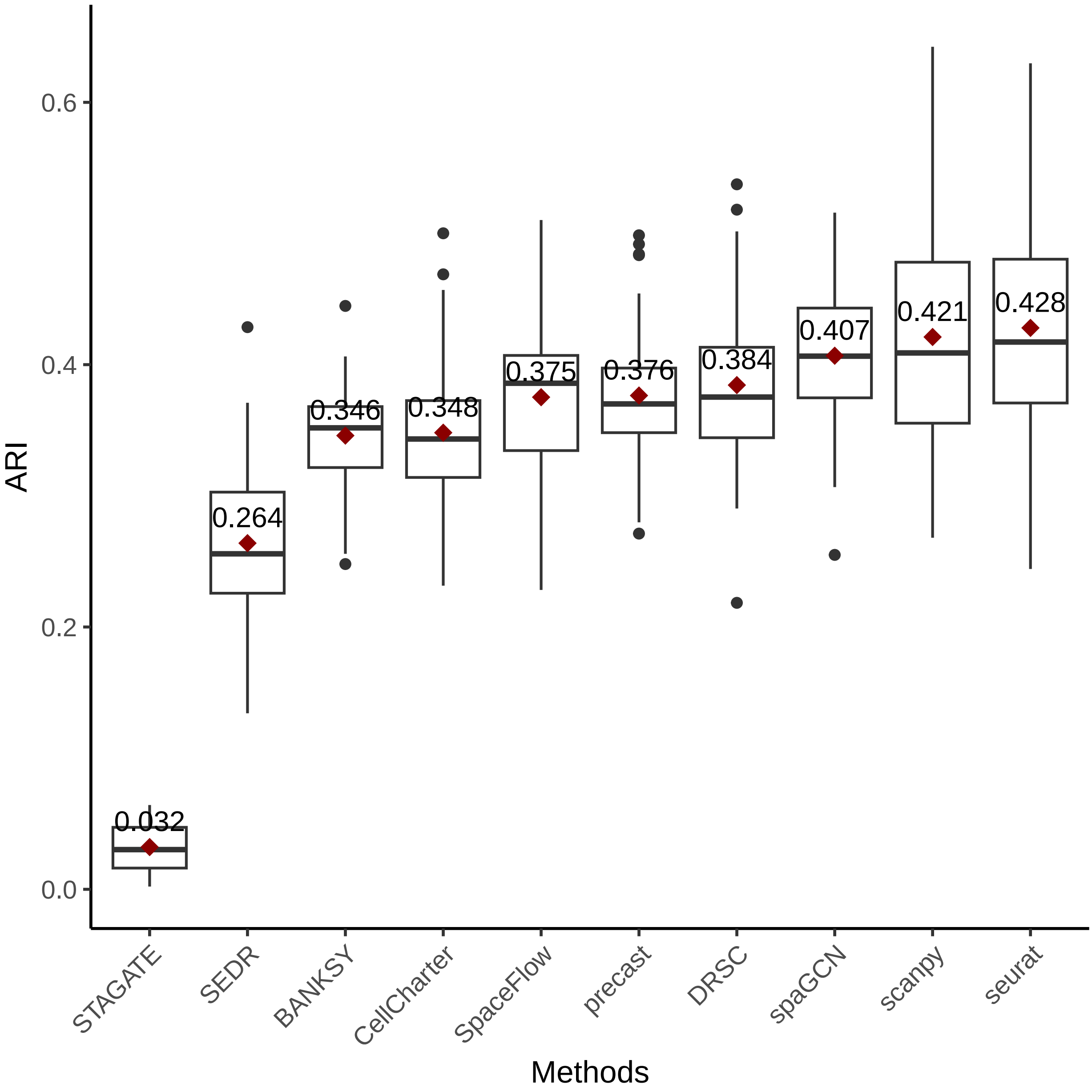

Figure S9: ARI results for Stereo-seq mouse embryo dataset, with mean value indicated in red square.

bioRxiv preprint doi: https://doi.org/10.1101/2025.06.23.660861; this version posted June 27, 2025. The copyright holder for this preprint (which was not certified by peer review) is the author/funder, who has granted bioRxiv a license to display the preprint in perpetuity. It is made available under aCC-BY 4.0 International license.

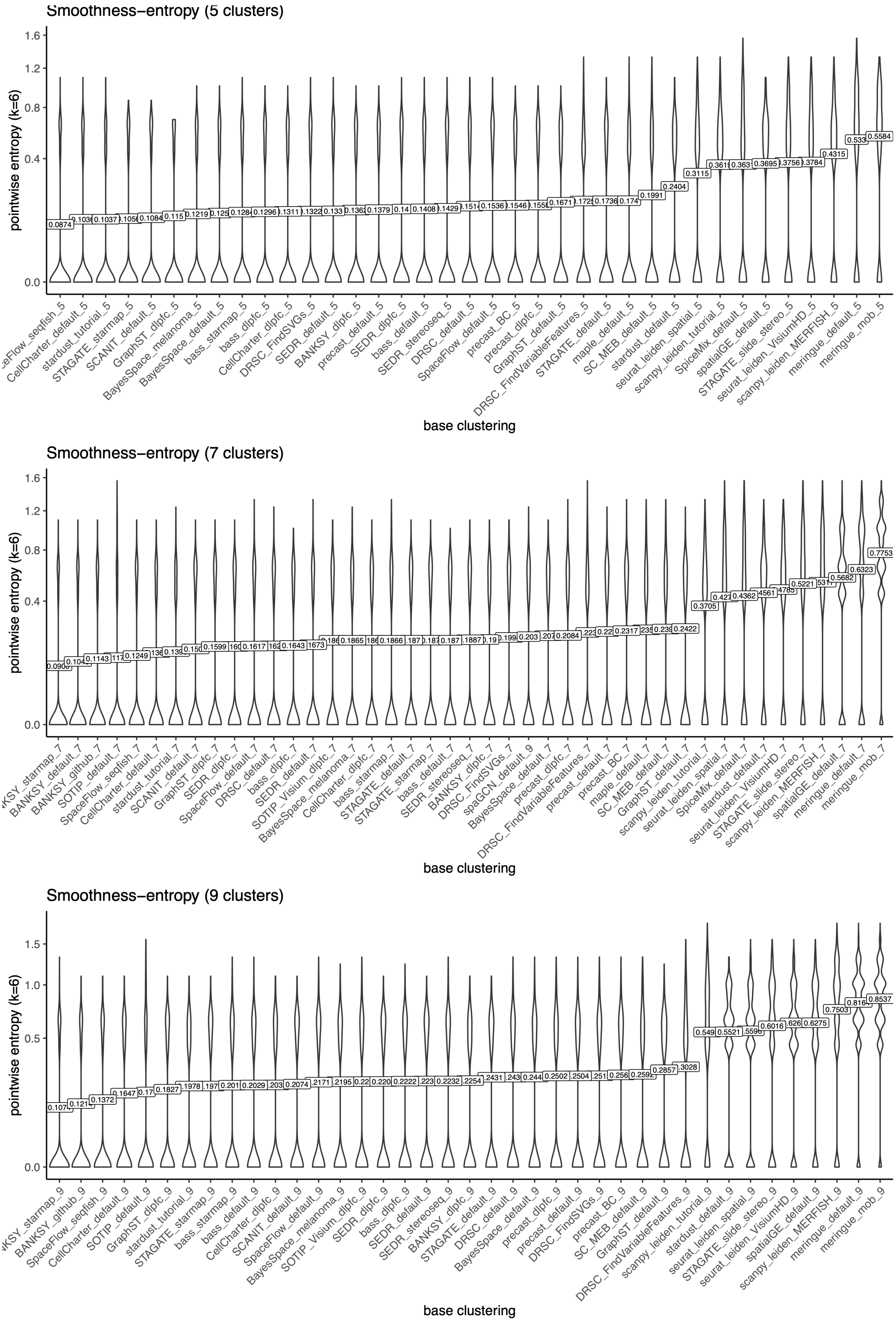

Figure S10: Distributions of smoothness entropy scores for all base clusterings of LIBD DLPFC slice 151673.

bioRxiv preprint doi: https://doi.org/10.1101/2025.06.23.660861; this version posted June 27, 2025. The copyright holder for this preprint (which was not certified by peer review) is the author/funder, who has granted bioRxiv a license to display the preprint in perpetuity. It is made available under aCC-BY 4.0 International license.

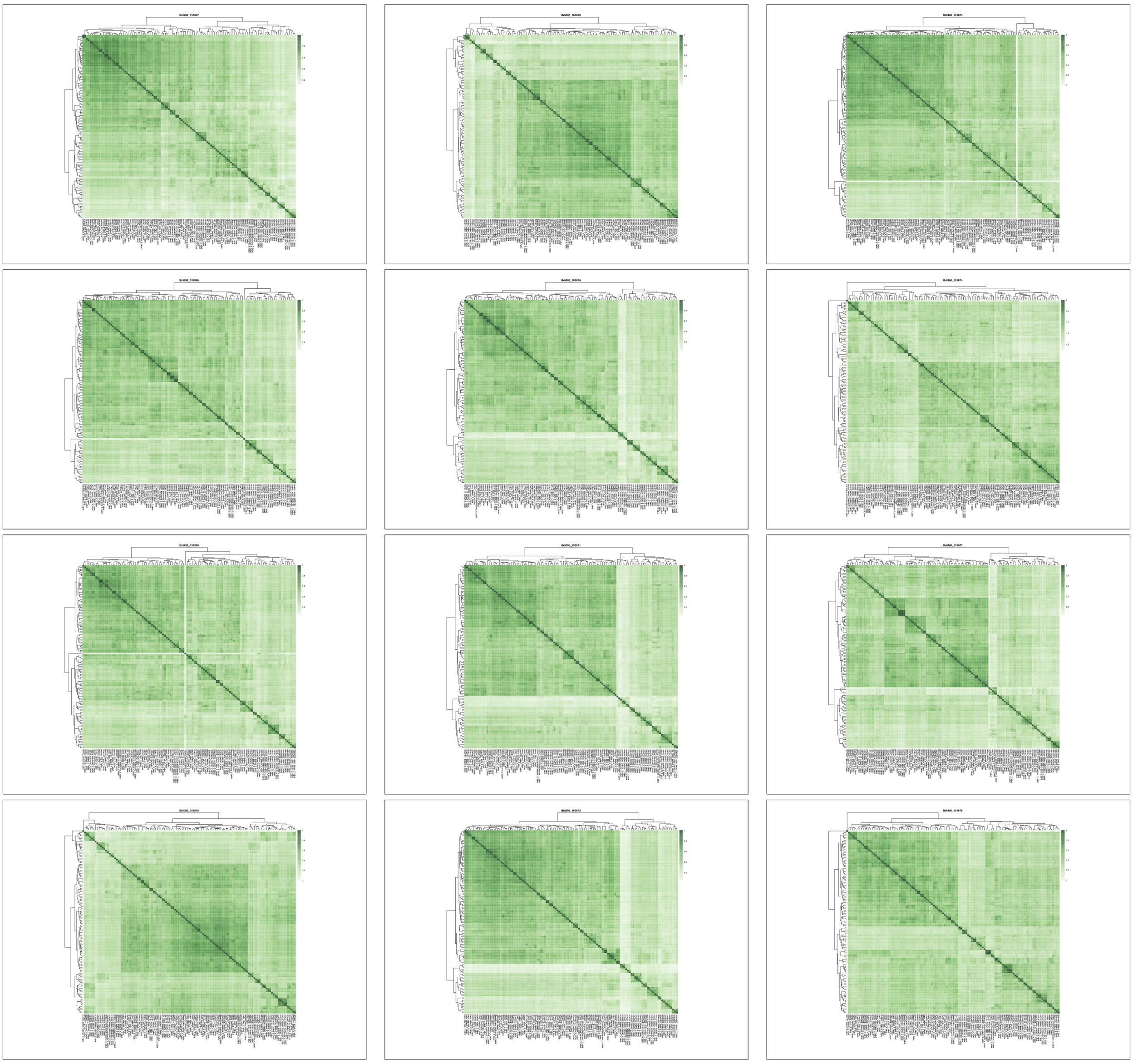

Figure S11: Heatmaps of pairwise ARIs between all base clusterings for each LIBD DLPFC slice. In all cases, we observed a block of concordant methods.

bioRxiv preprint doi: https://doi.org/10.1101/2025.06.23.660861; this version posted June 27, 2025. The copyright holder for this preprint (which was not certified by peer review) is the author/funder, who has granted bioRxiv a license to display the preprint in perpetuity. It is made available under aCC-BY 4.0 International license.

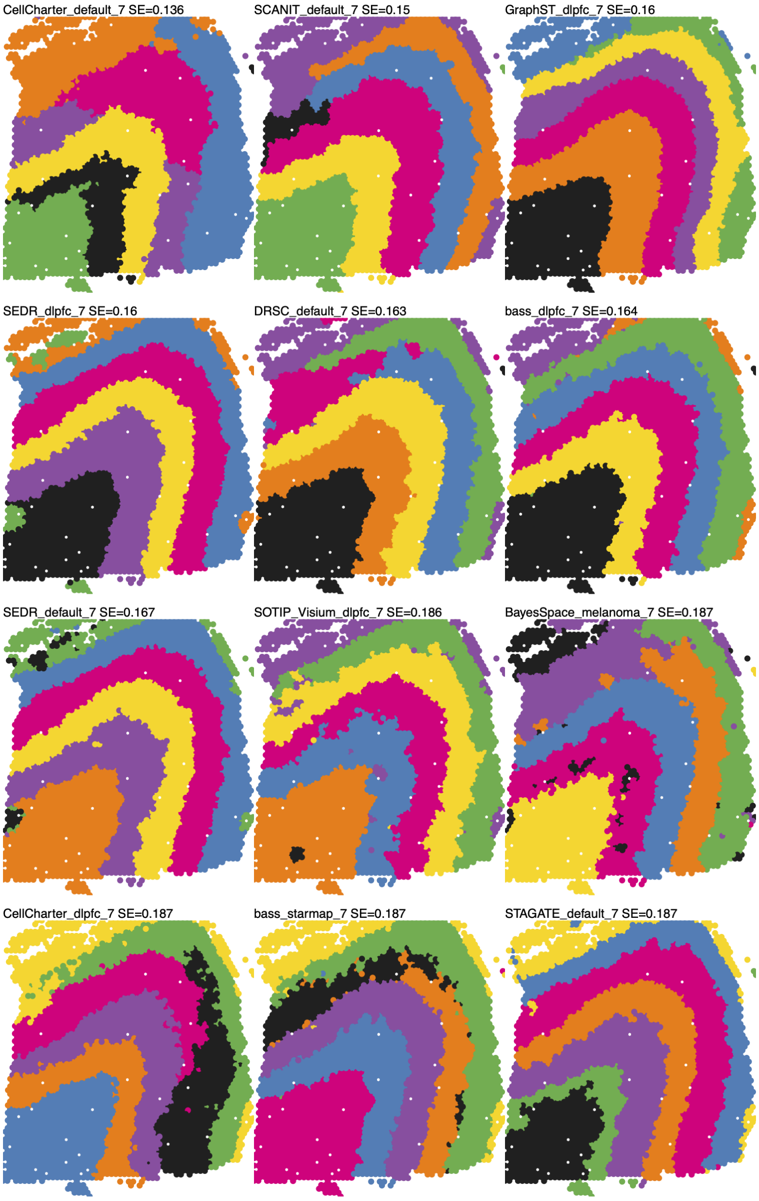

Figure S12: 12 selected base clusterings at 7 clusters to form consensus used for Figure 3B ("consensus_lca") (and represented in Figure 3D) for LIBD DLPFC slice 151673.

bioRxiv preprint doi: https://doi.org/10.1101/2025.06.23.660861; this version posted June 27, 2025. The copyright holder for this preprint (which was not certified by peer review) is the author/funder, who has granted bioRxiv a license to display the preprint in perpetuity. It is made available under aCC-BY 4.0 International license.

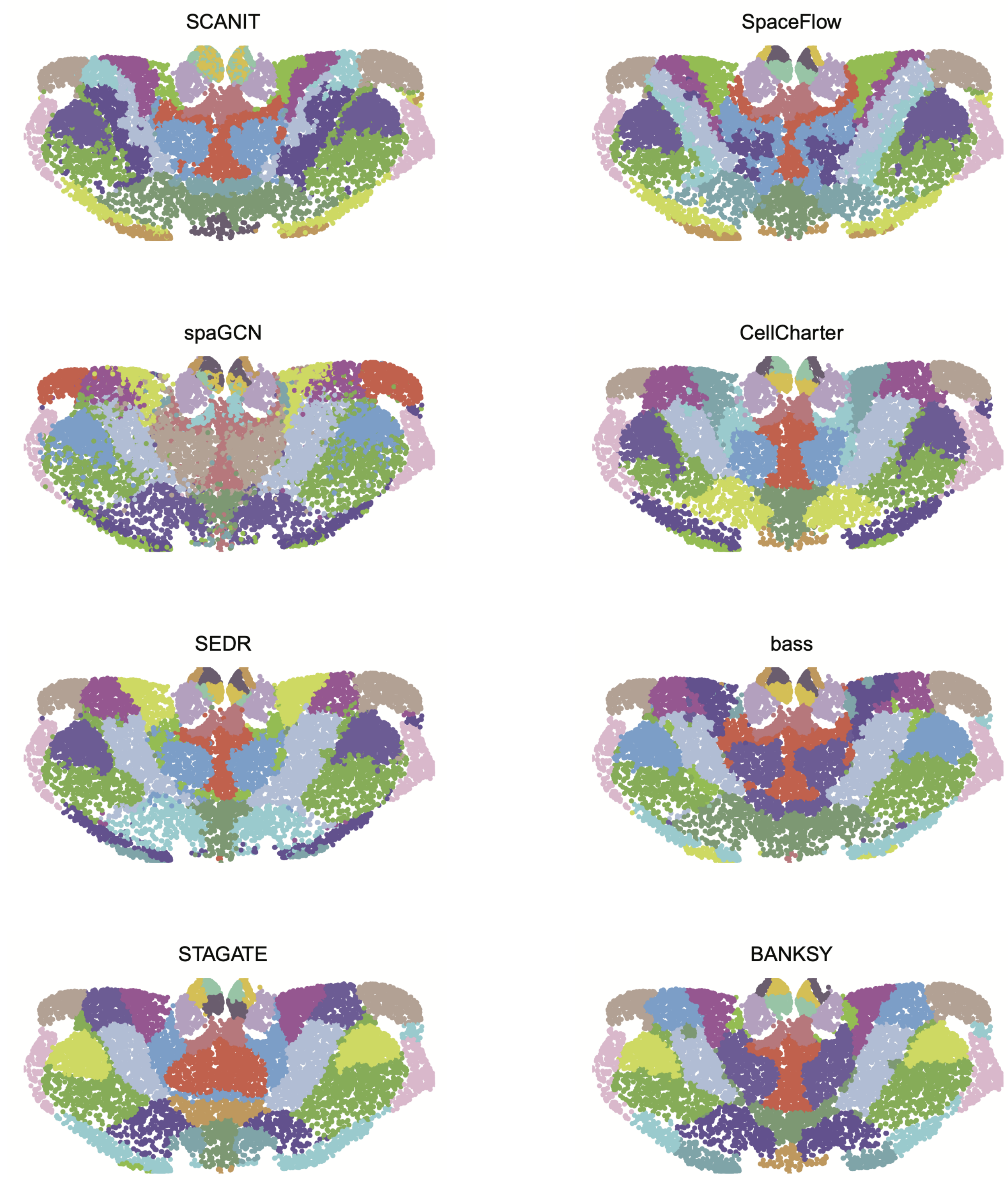

Figure S13: Clusters in the mouse thalamus generated from running 8 SAC methods with the cluster parameter set to 20.

bioRxiv preprint doi: https://doi.org/10.1101/2025.06.23.660861; this version posted June 27, 2025. The copyright holder for this preprint (which was not certified by peer review) is the author/funder, who has granted bioRxiv a license to display the preprint in perpetuity. It is made available under aCC-BY 4.0 International license.

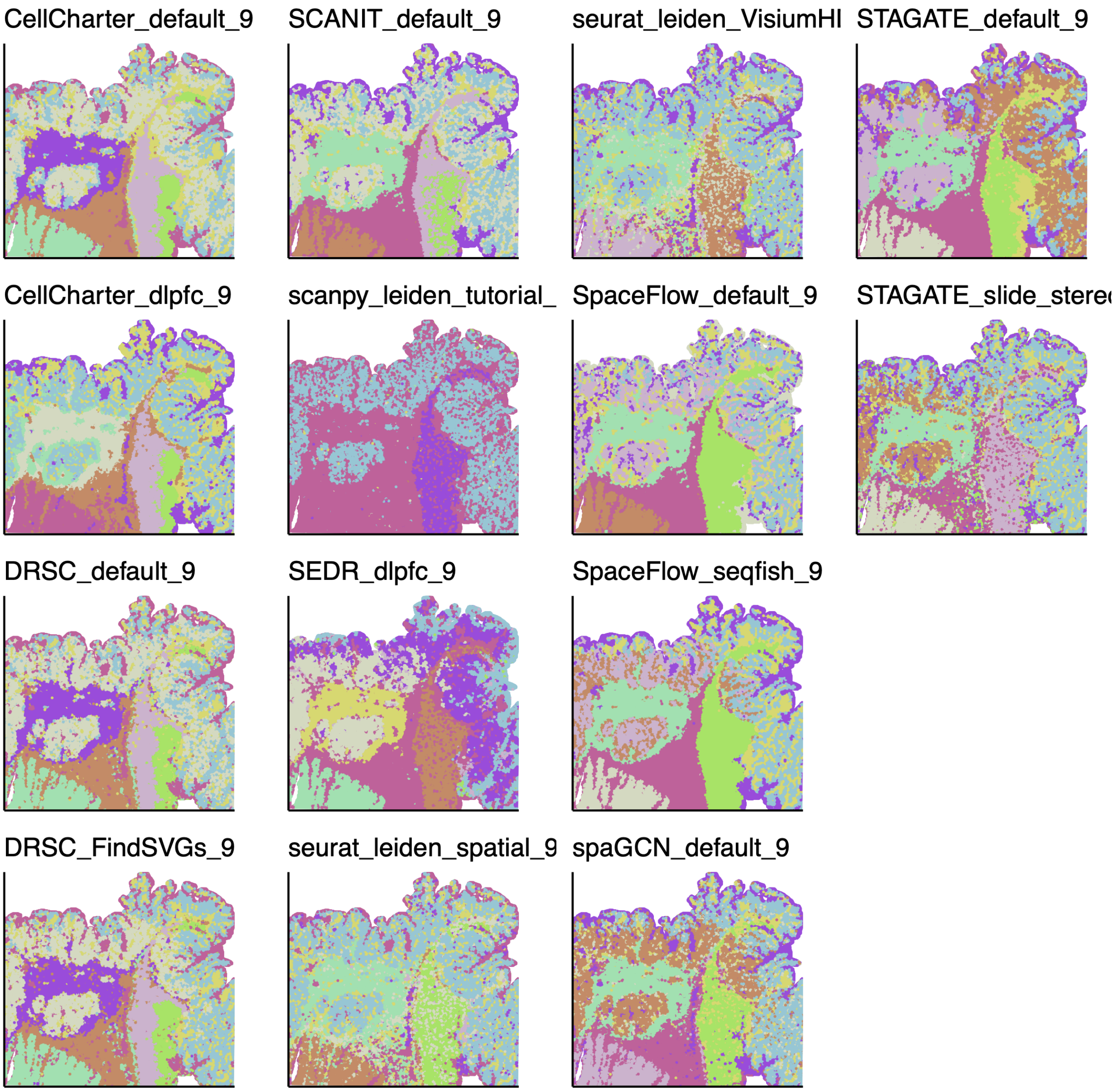

Figure S14: Base clusterings of full Visium HD cancer datasets with 9 clusters for pathologist evaluation.

bioRxiv preprint doi: https://doi.org/10.1101/2025.06.23.660861; this version posted June 27, 2025. The copyright holder for this preprint (which was not certified by peer review) is the author/funder, who has granted bioRxiv a license to display the preprint in perpetuity. It is made available under aCC-BY 4.0 International license.

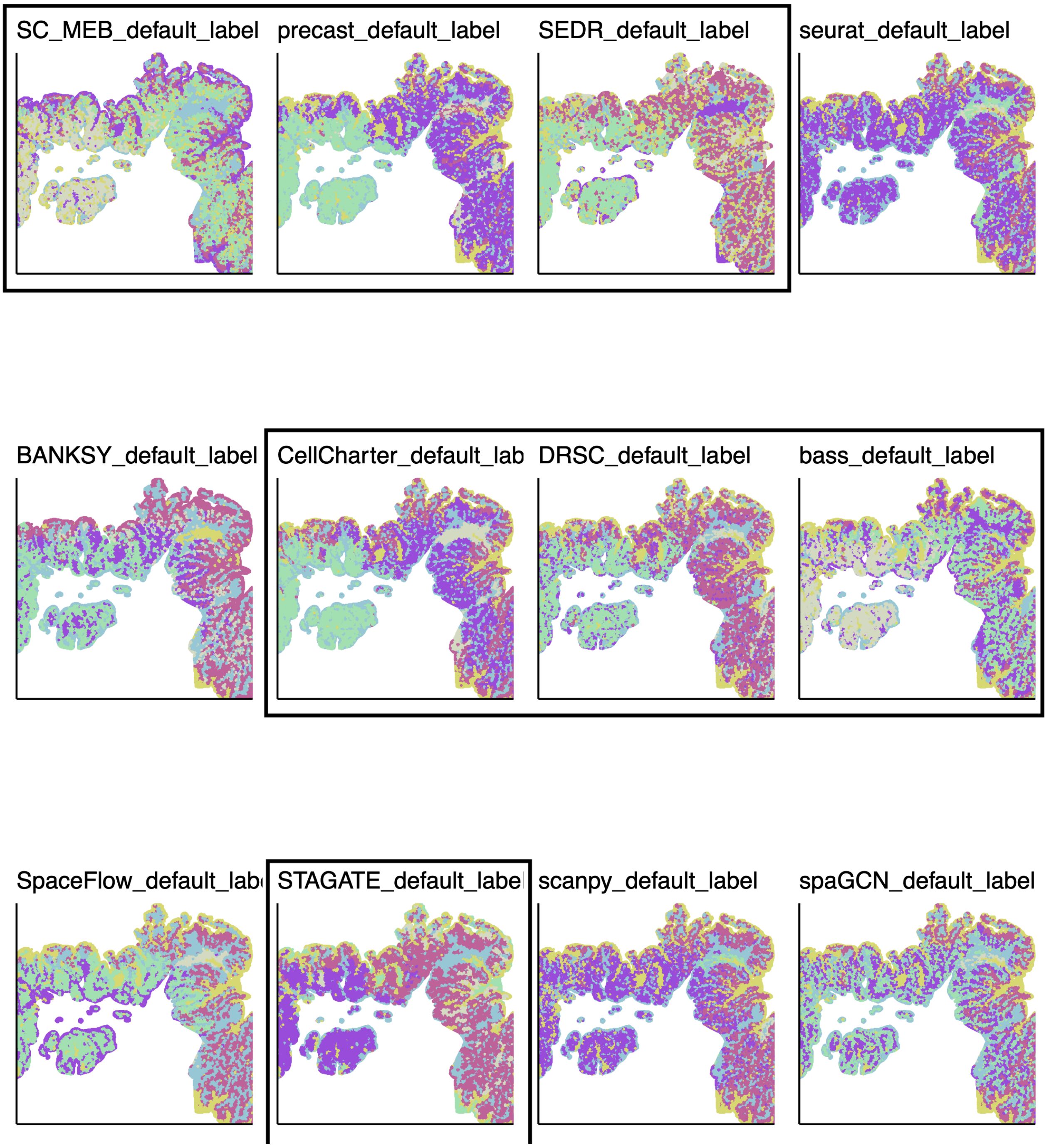

Figure S15: Base clusterings generated for subsetted Visium HD dataset, the instances selected for consensus are enclosed with black boxs.

bioRxiv preprint doi: https://doi.org/10.1101/2025.06.23.660861; this version posted June 27, 2025. The copyright holder for this preprint (which was not certified by peer review) is the author/funder, who has granted bioRxiv a license to display the preprint in perpetuity. It is made available under aCC-BY 4.0 International license.

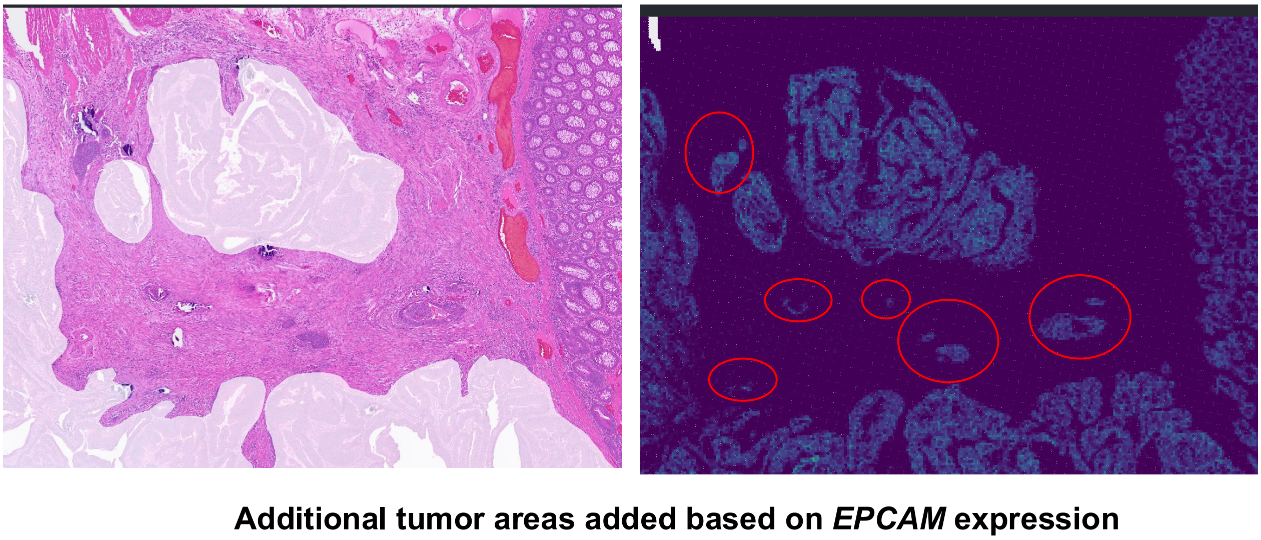

Figure S16: *EPCAM* expression used for fine annotation of the neoplasm. Circles in red were initially missed based solely on the HE

bioRxiv preprint doi: https://doi.org/10.1101/2025.06.23.660861; this version posted June 27, 2025. The copyright holder for this preprint (which was not certified by peer review) is the author/funder, who has granted bioRxiv a license to display the preprint in perpetuity. It is made available under aCC-BY 4.0 International license.

Supplementary Tables

Table S1: Overview of methods. A GitHub commit is only included for methods installed from GitHub without releases.

Name	Version (commit)	Year	Language	Citation
MAPLE	0.99.1 (b173e89)	2024	R	Jeon et al. (2024)
BANKSY	0.99.7 (dbda6fd)	2023	R (+ Python)	Singhal et al. (2023)
PRECAST	1.6.3	2023	R	Liu et al. (2023)
GraphST	1.1.1	2023	Python	Long et al. (2023)
SPICEMIX	0.0.71	2023	Python	Chidester et al. (2023)
CellCharter	0.2.0	2023	Python	Varrone et al. (2023)
STAGATE	1.0.1 (48ce7f8)	2022	Python	Dong & Zhang (2022)
scMEB	1.1	2022	R	Yang et al. (2022)
SpaceFlow	1.0.4	2022	Python	Ren et al. (2022)
DR-SC	3.3	2022	R	Liu et al. (2022)
SCAN-IT	0.1 (ebf3894)	2022	Python	Cang et al. (2022)
SOTIP	1.0 (d3b762c)	2022	Python	Yuan et al. (2022)
Stardust	2.0 (f1b5417)	2022	R	Avesani et al. (2022)
BASS	1.1.0.016 (37980c9)	2022	R	Li & Zhou (2022)
spatialGE	1.2.0.0000	2022	R	Ospina et al. (2022)
SpaGCN	1.2.7	2021	Python	Hu et al. (2021)
BayesSpace	1.10.1	2021	R	Zhao et al. (2021)
SEDR	1.0.0 (8c273af)	2021	Python	Fu et al. (2021)
MERINGUE	1.0 (ca9e2cc)	2021	R	Miller et al. (2021)
Giotto	4.0.0	2021	R (+ Python)	Dries et al. (2021)
Scanpy	1.10.0	2018	Python	Wolf et al. (2018)
Seurat	5.0.1	2018	R	Butler et al. (2018)

Table S2: Overview of method configurations.

bioRxiv preprint doi: https://doi.org/10.1101/2025.06.23.660861; this version posted June 27, 2025. The copyright holder for this preprint (which was not certified by peer review) is the author/funder, who has granted bioRxiv a license to display the preprint in perpetuity. It is made available under aCC-BY 4.0 International license.

Table S3: Overview of metrics.

Name	Requires GT labels	Uses spatial information	Language	Citation
Adjusted Rand Index (ARI)	yes	no	Python	Hubert & Arabie (1985)
Spatial chaos score (CHAOS)	no	yes	R	Shang & Zhou (2022)
Calinski-Harabasz	yes	no	Python	Calinski & Harabasz (2007)
Completeness	yes	no	Python	Rosenberg & Hirschberg (2007)
Davies-Bouldin	no	no	Python	Davies & Bouldin (1979)
Entropy	yes	no	Python	Shannon (1948)
Fowlkes-Mallows Index (FMI)	yes	no	Python	Fowlkes & Mallows (1983)
Homogeneity	yes	no	Python	Rosenberg & Hirschberg (2007)
Local Inverse Simpson's Index (LISI)	yes	no	R	Korsunsky et al. (2019)
Mathew's Correlation Coefficient (MCC)	yes	no	Python	Chicco & Jurman (2020)
Normalized Mutual Information (NMI)	yes	no	R	Strehl & Ghosh (2002)
Percentage of Abnormal Spots (PAS)	no	yes	R	Shang & Zhou (2022)
SpatialARI	yes	yes	R	Luo & Germain (2025)
V measure	yes	no	Python	Rosenberg & Hirschberg (2007)
Cluster specific silhouette	no	no	R	Rousseeuw (1987)
Domain specific F1	yes	no	R	Rijsbergen (1979)
Jaccard	yes	no	Python	Jaccard (1912)

Table S4: SpaceHack 2.0 participants.
